# Family Caregiver Burden and Neuropsychiatric Symptoms in Japanese Community‐Dwelling People With Alzheimer's Disease: A Cross‐Sectional Study Using a Web‐Based Questionnaire

**DOI:** 10.1111/psyg.70143

**Published:** 2026-02-13

**Authors:** Tomoyuki Nagata, Shunichiro Shinagawa, Shinichi Noto, Kentaro Yamato, Naoki Mori, Keisuke Onuki

**Affiliations:** ^1^ Center for Dementia‐Related Diseases Airanomori Hospital Kagoshima Japan; ^2^ Department of Psychiatry The Jikei University School of Medicine Tokyo Japan; ^3^ Faculty of Rehabilitation Science Niigata University of Health and Welfare Niigata Japan; ^4^ Medical Affairs Otsuka Pharmaceutical Co., Ltd. Tokyo Japan; ^5^ Department of Public Health, Graduate School of Medicine Juntendo University Tokyo Japan

**Keywords:** Alzheimer dementia, care services, caregiver burden, hyperactivity, Japan, psychomotor

## Abstract

**Background:**

Previous studies of community‐dwelling people with Alzheimer's disease (AD) have reported an association between the severe neuropsychiatric symptoms (NPS) of dementia and caregiver burden. We explored the current status of family caregiver burden, NPS of dementia, and care service usage in Japanese community‐dwelling people with AD.

**Methods:**

A web‐based questionnaire was administered to cohabitant family caregivers of community‐dwelling people with AD from 13 to 27 November 2023. Amongst 8108 participants registered in the panel data, 705 family caregivers (age: 19–79 years) were selected. Participants completed the Japanese version of the Neuropsychiatric Inventory‐Brief Questionnaire.

**Results:**

Family caregivers (*n* = 705) had a mean ± standard deviation (SD) age of 54.6 ± 11.5 years, 56.9% were male, and 84.0% cared for a parent or in‐law with AD. Patients with AD had a mean ± SD age of 84.2 ± 8.8 years; 26.2% were male, and 90.6% had NPS, including 73.4% with hyperactivity (agitation, disinhibition, irritability and aberrant motor behaviour). Mean ± SD caregiving time per week was higher for caregivers of patients with NPS versus without NPS (24.1 ± 22.1 vs. 17.6 ± 14.0 h). The dissatisfaction with nursing care support services was higher amongst caregivers of patients with NPS vs. without NPS. To manage hyperactivity, 11.3% of caregivers administered medication and 11.5% relocated patients to a calm environment; 16.6% of the caregivers had no way to cope. Amongst caregivers who responded ‘administer medication’ in response to hyperactivity, 32.1% had care staff or a medical provider come in and administer oral medication and 18.9% took the patient to a medical facility to receive an injection or intravenous treatment.

**Conclusions:**

Caregiving for AD patients with NPS (vs. without NPS) was associated with longer duration of caregiving, greater usage of nursing care services and dissatisfaction with nursing care support services.

## 1 Introduction

Over the long‐term progression of dementia, neuropsychiatric symptoms (NPS) occur in approximately 90% of community‐dwelling people with dementia [1]. NPS in people with dementia has been associated with an increased risk of early hospitalisation, poorer prognosis for both the patients and their caregivers' lifespans, and an increase in healthcare costs [2].

NPS include delusions, hallucinations, agitation/aggression, dysphoria/depression, anxiety, euphoria/elation, apathy/indifference, disinhibition, irritability/lability and aberrant motor behaviour and often manifest after the onset of dementia [3]. Hyperactivity, in particular, is a component of NPS that includes various symptoms (e.g., agitation, disinhibition, irritability or aberrant motor behaviour) and is related to a higher caregiver burden [4]. In a systematic review examining the association between NPS and caregiver burden, most caregivers were female and most (53.8%) were the patient's child or spouse (36%) [5]. Another study showed that caregiver burden depends on the patient's NPS profile, with female caregivers more likely to be overburdened [6].

Potentially harmful behaviour (PHB) by caregivers of patients with NPS may be associated with inappropriate care and may exacerbate the patients' NPS [7]. In a Japanese study of 133 pairs of outpatients with dementia and their caregivers amongst clinic‐based elderly people, the NPS symptoms of agitation, irritability, and aberrant motor behaviour, along with apathy, sleep and nighttime behavioural disturbances, and appetite and eating disorders, were associated with PHB [7]. In a study of family caregivers of patients with dementia receiving home nursing care services in Japan, 30% were found to have PHB, and the risk of having PHB was notably higher when the patient with dementia had behavioural disturbances [8]. The current situation of cohabitant family caregivers in Japan amongst those caring for patients with NPS of dementia is still unknown, despite the known risk of PHB. In a super‐ageing society like Japan, identifying unmet needs may be useful so caregivers can develop targeted support strategies and to inform healthcare policies, including new integrated services within the family home. Therefore, we surveyed the current situation in Japan to help address the issue of gaps in caregiver needs.

The present study aimed to reveal the status of caregiver burden in Japan, particularly the caregiver burden due to hyperactivity, and the use of caregiving services amongst community‐dwelling Japanese people with Alzheimer's disease (AD) using a web‐based questionnaire, a more accessible and efficient survey method than conventional mail‐based approaches.

## 2 Materials and Methods

### 2.1 Study Design

This study was a descriptive, cross‐sectional survey using a web‐based questionnaire. Participants answered screening questions, and respondents who met the eligibility criteria were asked to complete the main survey from 13 to 27 November 2023 [9]. The survey included questions regarding NPS caregiver burden, AD patient background, and status of antidementia and antipsychotic medications, all of which were answered by the caregiver participants.

The Research Ethics Committee of Otsuka Pharmaceutical Co. Ltd. approved the protocol (Reception number: 230928). The study adhered to the Declaration of Helsinki and adhered to Good Clinical Practise guidelines. All study participants provided written informed consent. This study was registered at the University Hospital Medical Information Network Clinical Trials Registry under the identifier number UMIN000053306.

### 2.2 Participants

Amongst all participants registered as disease monitors with Macromill Inc. Tokyo, Japan, those who met all the inclusion criteria and none of the exclusion criteria were included in this study. All participants received financial compensation from Otsuka Pharmaceutical Co. Ltd. (Tokyo Japan), for their involvement in this research. Rewards were paid based on the number of questions answered, with participants earning 2–82 points per question, equivalent to 1 yen per point.

The inclusion criteria for caregivers were ages 19–79 years; living with a close family member, such as a spouse, child, parent, sibling, grandparent or grandchild (including common‐law family members) with AD; and being the primary or partial caregiver of the family member with AD, based on self‐report.

### 2.3 Evaluation Items

Caregiver burden was assessed using the eight‐item short Japanese version of the Zarit Caregiver Burden Interview (J‐ZBI_8) [10]. The caregiver characteristics evaluated were age, sex, education, employment status, personal income, response when hyperactivity (including agitation, disinhibition, irritability, aberrant motor behaviour) occurs and satisfaction with nursing care support services.

In the patients with AD, the presence of NPS was determined using the Neuropsychiatric Inventory‐Questionnaire (NPI‐Q). The presence of NPS in a patient with AD was defined as the caregiver reporting a total NPI‐Q score greater than zero, indicating the presence of at least one of the 12 symptom domains on the NPI‐Q. Other background factors evaluated in the patients with AD were age, sex, antidementia medications, comorbidities, level of care required, medication taken when hyperactivity occurs, education, employment status, personal income, presence of relatives living with the patient with AD other than the caregiver, relationship with the caregiver, availability of caregiver support, hours of caregiving time received per week (by a family member and by a nursing care support service) and use of nursing care support service per week.

The certification of long‐term care needs was determined by a primary judgement made digitally and a secondary judgement made by several experts in health, medical care and welfare [11]. Additional details on the support levels needed are provided in Supporting Information Methods 1.

### 2.4 Statistical Methods

Frequencies were calculated for categorical variables, and summary statistics (mean, standard deviation [SD], quartiles and maximum and minimum values) were calculated for continuous data. Incorrect responses (multiple selection of rare diseases that affected family members living in the same household) were excluded. Missing values were not imputed. Statistical analyses were performed using Stata 18/MP8 (Stata Corp., College Station, Texas, USA), SAS 9.4 (SAS Institute Inc., Cary, North Carolina, USA) or Python version 3.10 (Python Software Foundation, Wilmington, Delaware, USA). Specifically, Stata and Python were used for general statistical analysis (excluding analyses involving the presence or absence of hyperactivity), whilst SAS was used exclusively for analyses related to the presence or absence of hyperactivity and for dataset quality control.

## 3 Results

### 3.1 Study Participants

**FIGURE 1:**
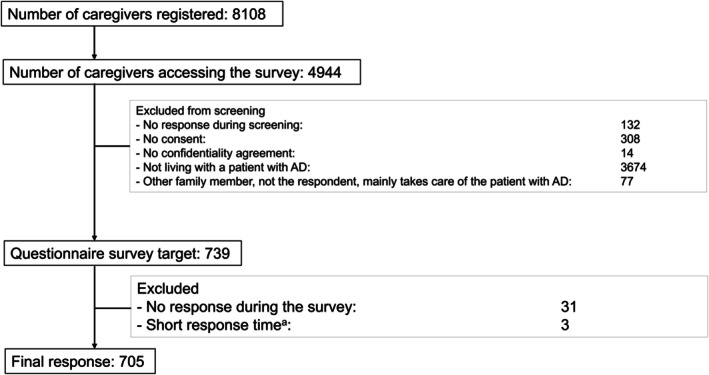
Participant flowchart from panel registration to final response. ^a^Based on the short‐response‐time respondent judgement criteria from Macromill Inc. AD, Alzheimer's disease.

Of 8108 caregivers registered, 4944 accessed the survey (Figure 1). In total, 4205 participants failed the screening, leaving 739 caregivers as the survey target. Completed surveys were obtained from 705 caregivers of patients with AD, of whom 639 (90.6%) were determined to have NPS and 66 (9.4%) did not.

The percentage of male caregivers was higher for patients without NPS than those with NPS (74.2% vs. 55.1%, respectively) (Table 1). In the overall population, caregivers' mean ± standard deviation (SD) age was 54.6 ± 11.5 years and was similar between groups with and without NPS. More than 80% of the caregivers cared for a parent or in‐law with AD. The mean ± SD J‐ZBI_8 score was twice as high amongst caregivers of patients with NPS than those caring for patients without NPS (16.2 ± 8.1 vs. 8.8 ± 7.7). The mean ± SD hours of caregiving time per week (by a family caregiver) was 25.7 ± 31.8 h/week for patients with NPS and 15.0 ± 25.4 h/week for those without NPS (Table 2).

**TABLE 1:** Background characteristics of patients with AD and their caregivers.

	*N*	Overall *N* = 705	With NPS *n* = 639	Without NPS *n* = 66
Caregiver sex, *n* (%)	705			
Male		401 (56.9)	352 (55.1)	49 (74.2)
Caregiver age, years, mean ± SD	705	54.6 ± 11.5	54.30 ± 11.5	57.7 ± 11.7
Relationship to the patient, *n* (%)	705			
Spouse/partner		39 (5.5)	33 (5.2)	6 (9.1)
Siblings and siblings‐in‐law		3 (0.4)	3 (0.5)	0 (0)
Parents and in‐laws		592 (84.0)	534 (83.6)	58 (87.9)
Grandparents and grandparents‐in‐law		65 (9.2)	64 (10.0)	1 (1.5)
Other		6 (0.9)	5 (0.8)	1 (1.5)
Primary caregiver, *n* (%)	705			
Respondent		428 (60.7)	391 (61.2)	37 (56.1)
Other family member		277 (39.3)	248 (38.9)	29 (43.9)
Patient age, years	705	84.2 ± 8.8	84.3 ± 8.8	82.9 ± 9.1
Patient sex, *n* (%)	705			
Male		185 (26.2)	170 (26.6)	15 (22.7)
Patient's last level of education, *n* (%)	705			
Common elementary school		41 (5.8)	39 (6.1)	2 (3.0)
Junior high school		196 (27.8)	178 (27.9)	18 (27.3)
High school		272 (38.6)	247 (38.7)	25 (37.9)
College of technology and junior college		79 (11.2)	69 (10.8)	10 (15.2)
Post‐secondary education institution, incl. university, college, etc.		95 (13.5)	87 (13.6)	8 (12.1)
Graduate school		4 (0.6)	4 (0.6)	0 (0)
Other		18 (2.6)	15 (2.3)	3 (4.5)
Requiring support or long‐term care, *n* (%)	705			
Requiring support level 1		42 (6.0)	35 (5.5)	7 (10.6)
Requiring support level 2		31 (4.4)	27 (4.2)	4 (6.1)
Requiring long‐term care level 1		162 (23.0)	150 (23.5)	12 (18.2)
Requiring long‐term care level 2		167 (23.7)	156 (24.4)	11 (16.7)
Requiring long‐term care level 3		122 (17.3)	113 (17.7)	9 (13.6)
Requiring long‐term care level 4		75 (10.6)	72 (11.3)	3 (4.5)
Requiring long‐term care level 5		63 (8.9)	49 (7.7)	14 (21.2)
Not applicable		31 (4.4)	26 (4.1)	5 (7.6)
Unknown		12 (1.7)	11 (1.7)	1 (1.5)
J‐ZBI_8, mean ± SD	705	15.5 ± 8.4	16.2 ± 8.1	8.8 ± 7.7
Antidementia drug use	705			
Donepezil		195 (27.7)	182 (28.5)	13 (19.7)
Galantamine		62 (8.8)	57 (8.9)	5 (7.6)
Rivastigmine		55 (7.8)	51 (8.0)	4 (6.1)
Memantine		114 (16.2)	107 (16.7)	7 (10.6)
Other		158 (22.4)	143 (22.4)	15 (22.7)
Unknown		185 (26.2)	157 (24.6)	28 (42.4)

Abbreviations: AD, Alzheimer's disease; J‐ZBI_8, eight‐item short Japanese version of the Zarit Caregiver Burden Interview; NPS, neuropsychiatric symptoms; SD, standard deviation.

**TABLE 2:** NPI‐Q score, caregiving time per week, availability of nursing care support service, and satisfaction with nursing care support service.

	Overall *N* = 705	With NPS *n* = 639	Without NPS *n* = 66	With hyperactivity *n* = 469	Without hyperactivity *n* = 236
NPI‐Q score					
Mean ± SD	7.2 ± 6.2	8.0 ± 6.1	—	9.6 ± 6.2	2.5 ± 2.4
Median	6	6	—	9	2
Q1, Q3	3, 10	3, 11	—	5, 13	0, 4
Min, max	0, 36	1, 36	—	1, 36	0, 12
Hours of caregiving time per week (by family caregiver^a^)					
Mean ± SD	24.7 ± 31.4	25.7 ± 31.8	15.0 ± 25.4	27.2 ± 33.3	19.6 ± 26.5
Median	14	15	5.5	15	10
Q1, Q3	5, 30	5, 30	1, 15	6, 30	3, 25
Min, max	1, 168	1, 168	1, 130	1, 168	1, 168
Availability of nursing care support service, *n* (%)					
With	518 (73.5)	473 (74.0)	45 (68.2)	350 (74.6)	168 (71.2)
Hours of caregiving time per week (by nursing care support service)					
*n*	518	473	45	350	168
Mean ± SD	23.6 ± 21.6	24.1 ± 22.1	17.6 ± 14.0	23.0 ± 20.8	24.7 ± 23.1
Median	20	20	14	20	20
Q1, Q3	9, 30	10, 30	7, 21	9, 30	10, 35
Min, max	1, 168	1, 168	1, 70	1, 168	1, 168
Caregiver satisfaction with nursing care support service, *n* (%)					
*n*	705	473	45	350	168
Quite satisfied	141 (27.0)	125 (26.4)	16 (35.6)	87 (24.9)	54 (32.1)
Somewhat satisfied	238 (46.0)	218 (46.1)	20 (44.4)	154 (44.0)	84 (50.0)
Undecided	92 (18.0)	83 (17.5)	9 (20.0)	70 (20.0)	22 (13.1)
Somewhat unsatisfied	35 (6.8)	35 (7.4)	0 (0.0)	31 (8.9)	4 (2.4)
Quite unsatisfied	12 (2.3)	12 (2.5)	0 (0.0)^b^	8 (2.3)	4 (2.4)

Abbreviations: NPI‐Q, neuropsychiatric inventory‐questionnaire; NPS, neuropsychiatric symptoms; Q, quartile; SD, standard deviation.

^a^ Family members within the second degree of kinship, including common‐law family members.

^b^ Nonparametric.

Regarding the characteristics of patients with AD, the mean ± SD age in the overall population was 84.2 ± 8.8 years and was similar between the group with and without NPS. Most patients with AD were female (73.8%) (Table 1). Amongst 639 patients with NPS, 469 (73.4%) had hyperactivity.

### 3.2 Antidementia Drug Use

The distribution of antidementia drug use was similar between the group with NPS and the group without NPS (Table 1). The use of donepezil and memantine was higher in the group with NPS versus without NPS (donepezil: 28.5% vs. 19.7%, respectively; memantine: 16.7% vs. 10.6%, respectively). The percentage of unknown prescriptions (those who responded, ‘I don't know’) was notably high in the group without NPS (42.4%). No statistical test was performed to compare the two groups.

### 3.3 Use of Nursing Care Support Services, Nursing Care Level Needed, and Satisfaction With Nursing Care Support Services

Nursing care support services were available for most patients (74.0% with NPS; 68.2% without NPS) (Table 2). The mean ± SD hours of caregiving time per week (by a nursing care support service) was 24.1 ± 22.1 h/week for patients with NPS and 17.6 ± 14.0 h/week for those without NPS, whilst caregiving time per week was 23.0 ± 20.8 h/week for patients with hyperactivity and 24.7 ± 23.1 h/week for patients without hyperactivity. The mean hours of caregiving per week for patients with NPS was greater than for those without NPS, whilst the mean hours of caregiving per week for patients with hyperactivity was greater than for those without hyperactivity.

Patients with NPS most commonly required nursing care level 2 (24.4%), and amongst patients without NPS, the most required nursing care level was 5 (21.2%) (Table 1). The required nursing care level distribution was wider for patients without NPS than for those with NPS. The percentage of patients who needed long‐term care level ≥ 1 was 86.0% for those with NPS and 75.4% for those without NPS. Although the present study's target population was limited to caregivers of patients diagnosed with AD, when asked about the level of nursing care needed, 9% of the caregivers of patients without NPS reported ‘not applicable’ or that they were uncertain about the category of care needed (response: I don't know) (Table 1). Caregivers of patients with NPS reported varying dissatisfaction with the nursing care support service, with 7.4% somewhat unsatisfied and 2.5% quite unsatisfied. In contrast, none of the caregivers of patients without NPS reported being somewhat or quite unsatisfied with the nursing care support service (Table 2).

### 3.4 Solution or Coping Strategy Used by Caregivers for Hyperactivity

When caregivers were asked, ‘How do you respond when the patient with AD exhibits aggressive statements or behaviour such as yelling, screaming, hitting others, throwing things, etc.?’ the most frequent answer was ‘Communicate in a calming manner’ (57.1%). Less frequent responses were ‘Administer medication’ (11.3%), ‘Relocate the patient to a calm environment to minimise irritation’ (11.5%) and ‘Physically restrain the patient’ (3.0%). About 16.6% of caregivers responded, ‘There is no way to cope with it’. In total, 53 (11.3%) caregivers responded that they administer medication when the patient with AD exhibits hyperactivity (Table 3). Of the caregivers who responded to hyperactivity by administering medication, 32.1% had care staff or a medical provider administer oral medication to patients with AD, and 18.9% stated that they took patients to a medical facility to receive an injection or intravenous treatment (Table 4).

**TABLE 3:** Caregiver response to occurrence of hyperactivity.

	*n* = 469
Communicate in a calming manner	268 (57.1)
Respond with the help of another caregiver	96 (20.5)
Administer medication	53 (11.3)
Relocate the patient to a calm environment to minimise irritation	54 (11.5)
Physically restrain the patient	14 (3.0)
There is no way to cope with it	78 (16.6)
Other	58 (12.4)

*Note:* Data are *n* (%).

Multiple choices were allowed.

**TABLE 4:** Who administers medication at the time of occurrence of hyperactivity.

	*n* = 53^a^
Respondent or another caregiver administers oral medication	46 (86.8)
Have care staff or medical provider come in and administer oral medication	17 (32.1)
Take patient to a medical facility to receive an injection or intravenous treatment	10 (18.9)
Other	1 (1.9)

*Note:* Data are *n* (%).

Multiple choices were allowed.

^a^ A total of 53 caregivers responded that their response to the occurrence of hyperactivity was ‘administer medication’.

## 4 Discussion

This web‐based survey was conducted amongst family members of people with AD who were registered as disease monitors in Macromill Inc. to investigate the current status of caregiver burden, NPS, and use of caregiving services in community‐dwelling Japanese people with AD. Although many countries prioritise policies for supporting families that care for their family members with dementia at home for as long as possible, the reality of caregiving often has not reached such ideal goals [12].

This survey of 705 family caregivers of patients with AD revealed that 90.6% of the patients experienced NPS. Caregivers for those with NPS were less likely to be male (55.1%) than those without (74.2%). Most caregivers were caring for a parent or in‐law. Caregiver burden (as measured by the J‐ZBI_8) was twice as high amongst caregivers of patients with NPS than those caring for patients without NPS. Nursing care support services were available for 74% of patients with NPS and 68% of those without. Patients with NPS received more caregiving hours and had higher long‐term care needs than patients without NPS. Caregivers reported varying dissatisfaction with nursing support for patients with NPS, whilst none reported dissatisfaction for those without NPS. About 17% of caregivers felt they had no way to cope with hyperactivity, highlighting the need for education on coping strategies.

In AD, caregiver burden has been reported to correlate with the quality of life of caregivers [13]. In patients with dementia, the severity of caregiver burden has been reported to be associated with NPS and caregivers' depressive states [14]. A Japanese study on health and well‐being showed lower utility scores (for healthcare resource utilisation) and SF‐36 health‐related quality of life scores for caregivers of patients with AD or dementia (*n* = 1302) compared with non‐caregivers (*n* = 53 758), indicating that the impact of burden on caregivers is significant [15]. An Iranian cross‐sectional study of 85 patients with AD used the NPI‐Q and the Caregiver Burden Inventory to assess NPS in patients with AD and its relationship to caregiver burden [16]. NPS, particularly hallucinations, aberrant motor behaviour, delusions and depression, were associated with greater caregiver burden, and apathy was the most common symptom in patients with AD. Scores in agitation, euphoria, apathy, disinhibition, irritability and aberrant motor behaviour amongst NPI sub‐items have also been found to be significantly higher with increased severity in dementia [17], and the most common symptoms of NPS were aberrant motor behaviour, agitation and irritability [18]. An assessment of caregiver psychological distress in Japan using the Kessler Psychological Distress scale score showed that the impact of verbal abuse and resistance to caregiving was particularly significant [19].

Taken together, those findings in previous studies reinforce the link between the presence of NPS and caregiver burden, emphasising the importance of understanding both the status of caregiver burden and NPS in community‐dwelling Japanese people with AD. However, there is little evidence on the conditions faced by caregivers of patients with AD in Japan. Thus, understanding the relationship between caregiver burden and NPS may help in developing targeted interventions that alleviate caregiver distress and improve outcomes and quality of life of people with AD in Japan.

The trend of prescribed medications was similar between patients with and without NPS; however, the use of antidementia drugs tended to be more common amongst those with NPS. This may be because the caregivers of patients with NPS may have a greater desire for active intervention (including treatment) for NPS than caregivers of patients without NPS.

The use of care services has been linked to benefits for caregivers of patients with dementia [20, 21, 22]. In Japan, the Long‐Term Care Insurance System was introduced in 2000 to provide home‐based and facility‐based care services for people aged ≥ 65 years or those aged ≥ 40 years with a disease requiring caregiving [23]. Caregivers of people with dementia use nursing care support services to cope with the stress associated with caregiving [24, 25].

A previous study in Taiwan reported that approximately 60% of caregivers used long‐term care services, and long‐term care service use increased as the frequency of NPS increased [26]. In the present study, the use of nursing care support services was higher for patients with NPS (74.0%) versus without NPS (68.2%), and the mean hours of caregiving per week (by a family caregiver and a nursing care support service) was notably higher for patients with NPS than without NPS. Although greater use of nursing care support services was observed amongst caregivers of patients with NPS, the actual service use does not necessarily equate to the level of need. Service utilisation may be influenced by structural limitations such as the availability of services, financial constraints, or caregivers' personal preferences. Therefore, whilst our findings suggest a higher burden in the NPS group, this should be interpreted with caution, as unmet needs were not directly assessed in this study.

In the present study, the mean hours per week of caregiving time in the NPS group was 25.7 ± 31.8 h by family caregivers and 24.1 ± 22.1 h by nursing care support services. This finding is consistent with a US study in which caregivers reported spending an average of 27.8 ± 26.1 h per week caring for persons with dementia [27].

Hyperactivity may reduce the use of nursing care support services by disrupting access to these care services or leading to refusal of the service itself. If hyperactivity can be managed, appropriate treatment or care should be considered to ease the caregiver's burden. Care services provide respite by reducing caregiving hours and demands, alleviating the intensity of behaviour problems, and supporting family caregivers in managing complex care needs [21]. In‐home long‐term care services, as provided in the Japanese system to support home‐based and facility‐based care for people with AD, can reduce the burden on caregivers. Thus, future studies should investigate the reasons why caregivers with NPS are not using such services. A wider distribution of the required nursing care level was observed for patients without NPS than for those with NPS. The group with NPS (i.e., patients were at a stage where cognitive decline was progressing) tended to fall within a narrower range of nursing care levels, with most patients requiring care levels of 1 to 3, where activities of daily living are relatively maintained, and fewer patients requiring a care level of 5, where activities of daily living are in decline. In contrast, in the group without NPS, the proportion of patients requiring a care level of 5 was higher than that of patients requiring lower care levels. These findings are important because activities of daily living in patients with AD have been reported to be factors associated with caregiver burden [28]. In the stage of AD where impairment of activities of daily living is mild to moderate, NPS are more noticeable and burden caregivers, in part because the physical status of patients is still maintained. However, in the moderate to severe stages of AD, where activities of daily living are further reduced, NPS symptoms may become less noticeable, suggesting a potential trade‐off.

NPS may be recognised by caregivers at a stage when activities of daily living are relatively well‐maintained, and treatment intervention should be provided at an early stage to reduce the burden on caregivers. Many caregivers of people with NPS expressed satisfaction with nursing care support services, even with the presence of NPS. The results suggest that factors other than management of symptoms can contribute to satisfaction with nursing care support. These results are also supported by previous findings of a modest correlation between symptom burden and satisfaction with care [29, 30]. However, many caregivers of people with AD and NPS felt somewhat dissatisfied or quite dissatisfied with nursing care support services. In addition to the management of symptoms, other reasons for dissatisfaction with nursing care support may include difficulty in coping with NPS or finding solutions for managing NPS, lack of services despite higher needs, and inadequate services due to NPS, but further investigation is required to confirm this.

Almost 17% of all caregivers answered that they had no way to cope with the occurrence of hyperactivity. Despite the lack of coping methods, it is necessary to identify why home care is required and issues related to promoting appropriate nursing care support services. Of the 53 caregivers who responded to the occurrence of hyperactivity by administering medication, 32.1% answered that they had care staff or a medical provider come and administer oral medication to the patient, and 18.9% responded that they would take the patient to a medical facility to receive an injection or intravenous treatment. Thus, some caregivers in this study relied on nursing care support or medical services for administering medication. Therefore, it might be necessary to develop methods to support caregivers in managing patients with hyperactivity, such as providing specialised training or simplifying medication regimens.

Notably, 3% of caregivers reported using physical interventions as a behavioural management strategy for hyperactivity. Although this percentage is low, the human rights of people with dementia must be carefully considered. Perspectives on dementia have changed over time, with physical interventions to mitigate hyperactivity increasingly seen as an unacceptable option. Instead, person‐centred care is becoming more prevalent [31].

A potential study limitation is that over 80% of the caregivers in this study cared for a parent or in‐law with AD. This may limit the generalizability of the findings, as the sample included very few caregivers who were spouses, siblings or grandchildren of patients with AD and who may experience different caregiving challenges. Although no broad generalisations can be made about the current situation in Japan, the results of this study are important because of the implications for families caring for parents and in‐laws at home, given that the number of nuclear family households is expected to increase in the future. The generalisability of the findings is also limited to Japan; the study findings may not apply to other countries with different cultural and social support systems. This survey did not adequately capture caregivers' health status, which may have resulted in population bias. Finally, as this was a descriptive study, it is not possible to establish causal relationships from the results.

Individuals with hyperactivity are generally expected to impose a greater caregiving burden and to have higher needs for care services. However, given the current situation in which home caregiving hours are increasing, further augmentation of service provision systems and consideration of additional medical interventions are warranted. Although caregivers of patients with and without NPS face similar challenges, there is notable concern for those caring for patients with AD and hyperactivity, as they may not be receiving adequate support. Caregiving for AD patients with NPS is associated with longer caregiving hours and greater use of nursing care support services. However, these caregivers reported a higher dissatisfaction with such services than those caring for patients without NPS. About 17% of caregivers reported feeling they had no way to cope with hyperactivity. This highlights a significant gap in support and a need to reduce caregiver burden and improve the quality of support services. To reduce caregiver burden and enhance satisfaction, measures should focus on caregivers' understanding of the disease, providing them with effective coping strategies and ensuring they receive the necessary support to manage patients' behaviours, such as hyperactivity, appropriately.

## Funding

This study was funded by Otsuka Pharmaceutical Co. Ltd., Tokyo, Japan.

## Ethics Statement

The protocol was approved by the Research Ethics Committee of Otsuka Pharmaceutical Co. Ltd. (Reception number: 230928), and the study was conducted in accordance with the Declaration of Helsinki and adhered to Good Clinical Practice guidelines.

## Consent

All study participants provided written informed consent.

## Conflicts of Interest

Dr. Nagata has previously received the following two grants: a Grant‐in‐Aid for Scientific Research [grant number 25893251] from the Ministry of Education, Culture, Sports, Science and Technology, Japan, and a Chiba health preventive fund from Chiba Foundation for Health Promotion and Disease Prevention. Dr. Nagata has not received any funding or speaker's honoraria from pharmaceutical companies. Dr. Shinagawa has received speaker and/or manuscript fees from Otsuka Pharmaceutical Co. Ltd., Kowa Company Ltd., Sumitomo Pharma Co. Ltd., Eisai Co. Ltd., Takeda Pharmaceuticals Company Limited, MSD, Lundbeck Japan K.K., and Viatris Pharmaceuticals LLC and advisory fees from Eisai Co. Ltd., Otsuka Pharmaceutical Co. Ltd., and Ono Pharmaceutical Co. Ltd. Dr. Noto has received speaker's honoraria from Otsuka Pharmaceutical Co. Ltd. Dr. Yamato, Dr. Mori and Dr. Onuki are employees of Otsuka Pharmaceutical Co. Ltd.

## Supporting information


**Data S1:** psyg70143‐sup‐0001‐Supinfo.docx.

## Data Availability

Research data are not shared.
