# Supplementary material for: Family Caregiver Burden and Neuropsychiatric Symptoms in Japanese Community‐Dwelling People With Alzheimer's Disease: A Cross‐Sectional Study Using a Web‐Based Questionnaire
Source: Psychogeriatrics. 2026 Feb 13;26(2):e70143. doi: 10.1111/psyg.70143 (PMC12904849; doi:10.1111/psyg.70143)
Supplement: Supplementary file 1 — Data S1: psyg70143‐sup‐0001‐Supinfo.docx. [file PSYG-26-0-s001.docx]

**Supporting Information**

**Supporting Methods 1.** Determination of the levels of support needed

Based on five categories (direct life support, indirect life support, neuropsychiatric symptom-related activities, functional training-related activities, and medical-related activities), the standard hours for certification of long-term care needs were calculated, and the total of these hours and the dementia allowance were used to determine the level of support required: (1) support level 1, the standard time for long-term care certification is 25 to 32 minutes or an equivalent condition; (2) support level 2/long-term care level 1, 32 to 50 minutes or equivalent; (3) long-term care level 2, 50 to 70 minutes or equivalent; (4) long-term care level 3, 70 to 90 minutes or equivalent; (5) long-term care level 4, 90 to 110 minutes or equivalent; and (6) long-term care level 5, ≥110 minutes or equivalent.

Patients were considered independent (not applicable) if the patient could perform basic activities of daily living such as walking and getting up on his/her own, taking his/her medicine, and using the telephone. Patients were considered to require support if they could perform most of the basic activities of daily living themselves but required some assistance from a family caregiver (not from a nursing care support service). Patients were considered to require long-term care if the patient was unable to perform basic activities of daily living on his/her own and required some assistance from a nursing care support service.

Patients requiring long-term care were further classified as follows: requiring long-term care level 1, defined as a condition in which the patient’s ability to perform activities of daily living has deteriorated further from requiring support and partial long-term care is needed; requiring long-term care level 2, in addition to requiring long-term care level 1, partial nursing care is required for activities of daily living; requiring long-term care level 3, compared with requiring long-term care level 2, there is a significant decline in terms of both activities of daily living and instrumental activities of daily living, and almost total nursing care is required; requiring long-term care level 4, in addition to the requiring long-term care level 3, the patient’s ability to move is further reduced and it becomes difficult for the patient to lead daily life without nursing care support; and requiring long-term care level 5, the patient’s ability to perform activities of daily living is even worse than in those requiring long-term care level 4, and it is almost impossible for the patient to carry out daily living without nursing care support.
